# Enhanced exposure and visualization in splenic flexure mobilization with comparable perioperative outcomes: experience with Artisential^®^ during laparoscopic low anterior resection

**DOI:** 10.1007/s10151-025-03188-2

**Published:** 2025-12-29

**Authors:** E. Cho, H. S. Ryu, J.-S. Kim, S.-J. Baek, J.-M. Kwak, J. Kim

**Affiliations:** 1 Division of Colorectal Surgery, Department of General Surgery, Korea University Anam Hospital, 73 Goryeodae-ro, Seongbuk-gu, Seoul, 02841 Republic of Korea; 2 Korea University College of Medicine, 73 Goryeodae-ro, Seongbuk-gu, Seoul, 02841 Republic of Korea

**Keywords:** ARTISENTIAL, Articulation, Colorectal surgery, Laparoscopic surgery, Low anterior resection, Splenic flexure

## Abstract

**Background:**

For many surgeons performing laparoscopic colectomies, splenic flexure mobilization (SFM) remains one of the most technically challenging phases. To resolve challenges in laparoscopic SFM, we utilized Artisential^®^, a line of articulated laparoscopic instruments (ALI), to gain more freedom in traction and enlarge the visualized working space. We developed a study to demonstrate how Artisential^®^ allowed for a more efficient usage of surgical space during splenic flexure mobilization without surgical quality.

**Methods:**

This study consisted of two parts. First was a comparative analysis of dead space shown on screen during surgery with and without Artisential^®^ usage. Video recordings of nine consecutive laparoscopic low anterior resections (LAR) performed by a single surgeon using an Artisential^®^ grasper in the left (nondominant) hand were chosen as the experimental group. Among 43 LAR cases performed by the same surgeon in the previous year without the Artisential^®^, 9 cases most similar to the control were chosen by propensity score matching (PSM) of sex, age, distance from the anal verge, and preoperative chemoradiotherapy status. We compared the two groups in terms of average operation duration, postoperative complication severity, and the number of lymph nodes harvested.

**Results:**

Using an Artisential^®^ grasper for traction for splenic flexure mobilization during laparoscopic low anterior resections increased screen visualization by 11.8% compared with using conventional laparoscopic graspers. Length of operation, severity of postoperative complications, and number of harvested lymph nodes were comparable in both modalities.

**Conclusions:**

Angulated traction was utilized for splenic flexure mobilization in laparoscopic low anterior resections using a grasper from Artisential^®^, a line of articulated laparoscopic instruments. The surgeon was able to create a significantly larger working field and better exposure of target structures. This implementation did not affect operation time, recovery, or specimen integrity.

## Introduction

Laparoscopic surgery, with shorter hospital stay and less wound-related complications, has now become a mainstay in colorectal cancer surgery [1]. Among the various phases of laparoscopic colectomies, splenic flexure mobilization (SFM) is regarded as a particularly technically complex procedure. The splenic flexure (SF), the highest portion of the colonic tract, is sharply angulated and variably attached to its neighboring structures [2]. Such characteristics limit the surgeon’s view in dissection, even during laparoscopy [3].

Artisential^®^, a line of articulated laparoscopic instruments (ALI), provides 360-degree motion with its two joints. An Artisential^®^ grasper’s grasping action is achieved by pinching the thumb and index finger. It also has a locking feature that maintains the jaw’s articulated angle [4]. During our laparoscopic low anterior resections, we found these features to be particularly beneficial for SFM. They enabled the surgeon to manage the left colon mesentery with accuracy from various angles and depths, increasing the visible area of structures on screen. Additionally, the surgeon’s use of the ALI reduced the workload on the assistant surgeon, who typically lifts the left colon mesentery in an antigravity manner during SFM.

In this paper, we aimed to demonstrate the efficacy of using Artisential^®^ during the SFM phase in laparoscopic low anterior resection (LAR) by quantifying changes in two-dimensional screen usage. We also conducted a propensity score-matched comparison of LARs performed with and without an Artisential^®^ grasper to verify that its use does not compromise operative outcomes.

## Methods

This study, involving laparoscopic low anterior resections with routine full splenic flexure mobilization by a single surgeon (J.K.) performed from March to December 2023, comprised two parts. First, we graphically depicted the benefits of traction with an Artisential^®^ grasper during SFM (medial inframesocolic approach) using video recordings of nine LARs. In addition, gained surgical working space by using an Artisential^®^ grasper was quantified based on two-dimensional views captured from the surgical footage. When working to enter the lesser sac, a conventional laparoscopic dissector in the surgeon’s left hand held up the edge of the left colon mesentery. The fenestrated Artisential^®^ grasper was then introduced into the surgical field to perform the same movement while the laparoscope and other instruments, including those used by the assistant surgeon, were held still. Screenshots of the footage were taken at moments just before and after application of the Artisential^®^ grasper.

**Fig. 1:**
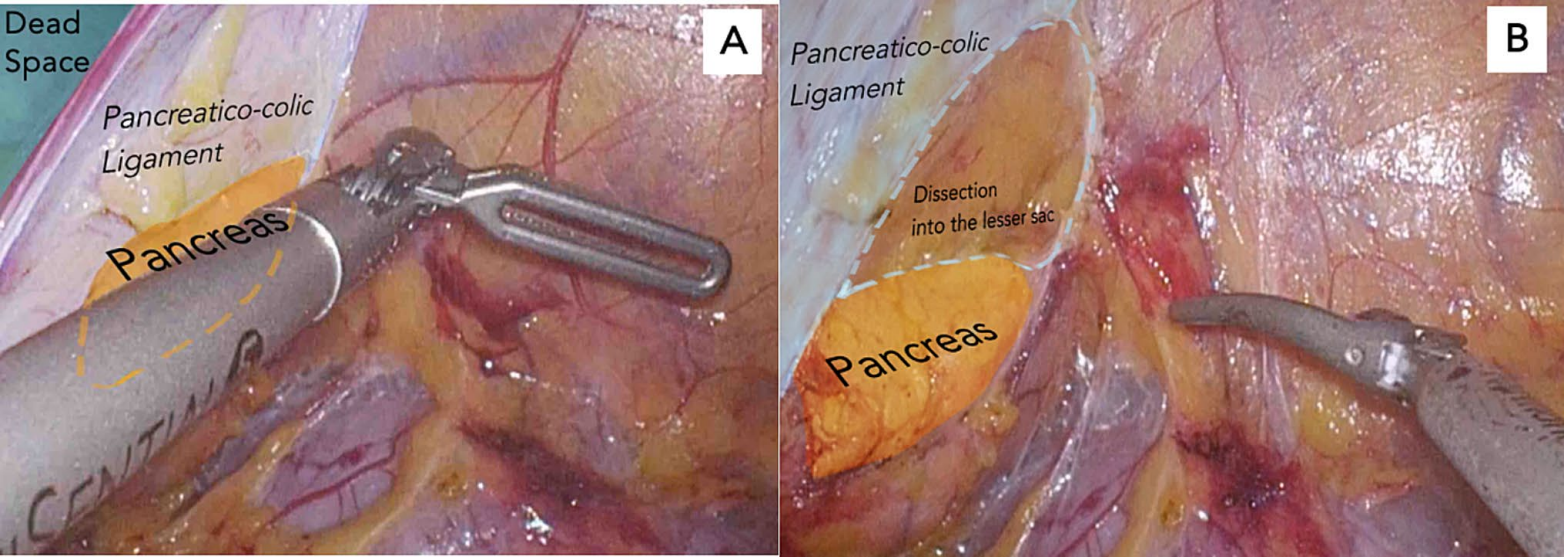
**A** “Dead space” above the left colon mesentery before angulated traction. **B** After using the Artisential^®^ grasper, the screen shows no unused space and only structures of interest

Two-dimensionally measured areas were as follows: (1) the entire screen and (2) “dead space” above the left colon mesentery, visible but not being operated on. “Screen utilization (%)” was defined as {(1) − (2)} ÷ (1) × 100 (%). Such calculations were done for each of the nine cases before (A) and after (B) Artisential^®^ use (Fig. 1). All calculations were done in pixels using Adobe Photoshop, version 25.0, Adobe Inc., San Jose, CA, USA.

The second part of the study compared perioperative outcomes of LARs performed with and without the Artisential^®^ fenestrated grasper. We utilized propensity score matching (PSM) to pair the 9 LARs performed with Artisential^®^ to 37 LARs performed in the previous year without Artisential^®^, in a 1:2 ratio. The nearest neighbor matching method without a caliper was applied. The criteria for PSM included sex, age, tumor distance from the anal verge, and whether the patient underwent preoperative chemoradiation. Each criterion weighted equally.

Compared perioperative outcomes were operation time, length of hospital stay, complications severity, and the number of harvested lymph nodes. Complication severity was graded using the Clavien–Dindo classification [5], with the highest grade observed in each case assigned. Shapiro–Wilk test revealed operation time and length of hospital stay to be normally distributed but not the other parameters. Hence, independent samples *t*-test was used for operation time and hospital stay, and Mann–Whitney *U* test for number of harvested lymph nodes and complication severity. All analyses were performed using Stata 18, StataCorp LLC, College Station, TX, USA.

Exclusion criteria for both groups were: T4b cases requiring extracolonic organ resection; LAR cases not needing full splenic flexure mobilization; patients with a history of colectomy; and open conversion cases.

This study was approved by the Korea UniversityInstitutional Review Board (IRB) (approval no. 2024AN0002), following the ethical standards of the Declaration of Helsinki.

## Results

### Screen Utilization Facilitated by the Artisential^®^ Grasper

On average, the surgical area visualized prior to traction with an Artisential^®^ grasper constituted 86.4% of the entire screen (range 77.0–96.7%). Following the application of Artisential^®^ for angulated traction, 98.2% (range 95.8–100%) of the screen displayed the surgical field of interest (an 11.8% increase in screen utilization). The calculated areas of utilization are detailed in Table 1.

**Table 1:** Screen utilization before and after Artisential^®^ usage

	Total area (pixels)	Before Artisential^®^ use			After Artisential^®^ use			Increased utilization (B–A) (%)
		Wasted (pixels)	Used (pixels)	Utilized (A) (%)	Wasted (pixels)	Used (pixels)	Utilized (B) (%)	
1	281,940	28,769	253,171	89.8	9502	272,438	96.6	6.8
2	241,326	38,881	202,445	83.9	2410	238,916	99.0	15.1
3	242,544	21,728	220,816	91.0	10,141	232,403	95.8	4.8
4	241,610	43,518	198,092	82.0	3335	238,275	98.6	16.6
5	240,500	35,828	204,672	85.1	25	240,475	100.0	14.9
6	240,870	16,950	223,920	93.0	9543	231,327	96.0	3.1
7	239,936	55,114	184,822	77.0	1675	238,261	99.3	22.3
8	241,980	51,222	190,758	78.8	4861	237,119	98.0	19.2
9	240,018	7815	232,203	96.7	0	240,018	100.0	3.3

### Propensity Score-Matched Comparison of Perioperative Outcome of Laparoscopic Low Anterior Resections with and without Artisential^®^ Use

After 1:2 matching, the 18 matched controls constituted a more balanced cohort than the original 37 controls. The standardized mean difference (SMD) improved from −1.16 to −0.20 for age, and from 0.15 to −0.04 for distance from the anal verge, respectively (Table 2).

**Table 2:** Comparison of covariates before and after propensity score matching

Variables for PSM	Artisential^®^ (*n* = 9)	Without Artisential^®^ (*n* = 18)		SMD	
		Prematch	Postmatch	Prematch	Postmatch
Age, mean (SD)	49.67 (11.14)	62.16 (10.34)	51.50 (7.47)	−1.16	−0.20
Sex (proportion, male)	0.50	0.51	0.51	−0.36	−0.33
Distance from the AV (cm) (mean (SD))	11.03 (4.66)	10.21 (6.04)	11.23 (6.41)	0.15	−0.04
PCRT (yes, proportion)	0.11	0.38	0.67	− 0.64	−1.35

*SD* standard deviation, *AV* anal verge, *SMD* standardized mean difference, *PCRT* preoperative chemoradiotherapy

The Artisential^®^ group and the propensity score-matched controls showed no difference in operation time (average 202.22 and 208 min, respectively) (*p* = 0.706), length of postoperative hospital stay (average 7.67 and 8.75 days, respectively) (*p* = 0.5), or complication severity (median grade 1 for both groups) (*p* = 0.622). The number of harvested lymph nodes in the final pathology report also did not differ between the two groups (21 and 23, respectively) (*p* = 0.801) (Table 3).

**Table 3:** Perioperative outcome with and without Artisential^®^

Variable	Group	Mean or median	SD	Minimum	Maximum	Test statistic	*p*-Value
Operation time (min)	Artisential^®^	201.22	27.68	158	256	−0.387	0.706
	Control	208	32.83	180	255		
Hospital stay (days)	Artisential^®^	7.67	1.8	5	11	−0.961	0.357
	Control	8.75	2.06	6	11		
Complication severity (Clavien–Dindo)	Artisential^®^	1	0.33	1	2	15.5	0.622
	Control	1	0.5	1	2		
Lymph nodes	Artisential^®^	21	5.96	13	34	16	0.801
	Control	23	4	21	29		

*SD* standard deviation

## Discussion

In our case series of laparoscopic low anterior resections, we found that angulated traction using an Artisential^®^ grasper significantly improved space utilization during splenic flexure mobilization. No significant differences in operation time, length of postoperative stay, or complication severity were observed between procedures performed with and without Artisential^®^, supporting its operative safety. Additionally, the equivalent number of harvested lymph nodes confirmed it does not compromise oncologic outcome.

As in previous studies assessing new techniques or instruments [6–8], we measured perioperative outcomes to evaluate the instrument’s safety during the surgeon’s adaptation period. We also included the number of harvested lymph nodes as a parameter to gauge the instrument’s appropriateness for cancer surgeries. International guidelines recommend a minimum number of harvested lymph nodes to ensure an oncologically adequate colon cancer resection [9, 10]. This is why the number of harvested lymph nodes is often used as a parameter to validate new surgical techniques [11, 12].

We chose the SFM phase to show the benefits of Artisential^®^ for two main reasons. First, among different phases of LAR, we saw that Artisential^®^ grasper usage had the greatest impact on this phase. Secondly, the minimal camera movement during the medial inframesocolic approach for SFM made it easy to visually depict the benefits of Artisential^®^.

**Fig. 2:**
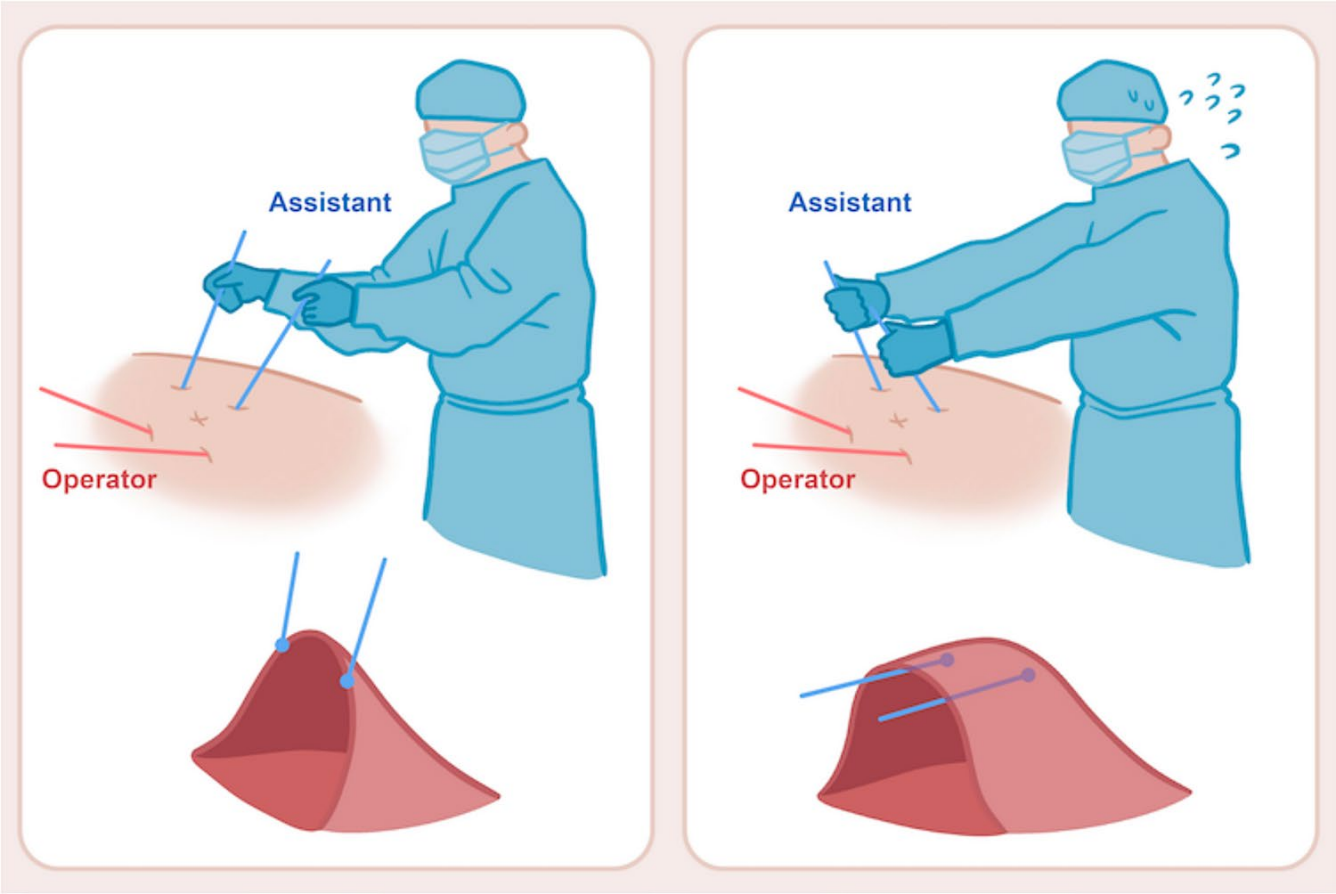
Schematic drawing of surgical working space created by assistant surgeon. The left shows assistant surgeon holding up the colon mesentery. The right shows assistant using leverage at the port sites to uplift the colon mesentery

The Artisential^®^ grasper enabled the surgeon to create an 11.8% larger two-dimensional working space compared with a conventional laparoscopic grasper. Before Artisential^®^, our typical LAR surgeries involved the assistant surgeon lifting the mesentery or using port sites to tent up the mesentery (Fig. 2). Although this method is acceptable, it demands considerable effort from the assistant and may still not offer an adequately large and well-visualized working area.

**Fig. 3:**
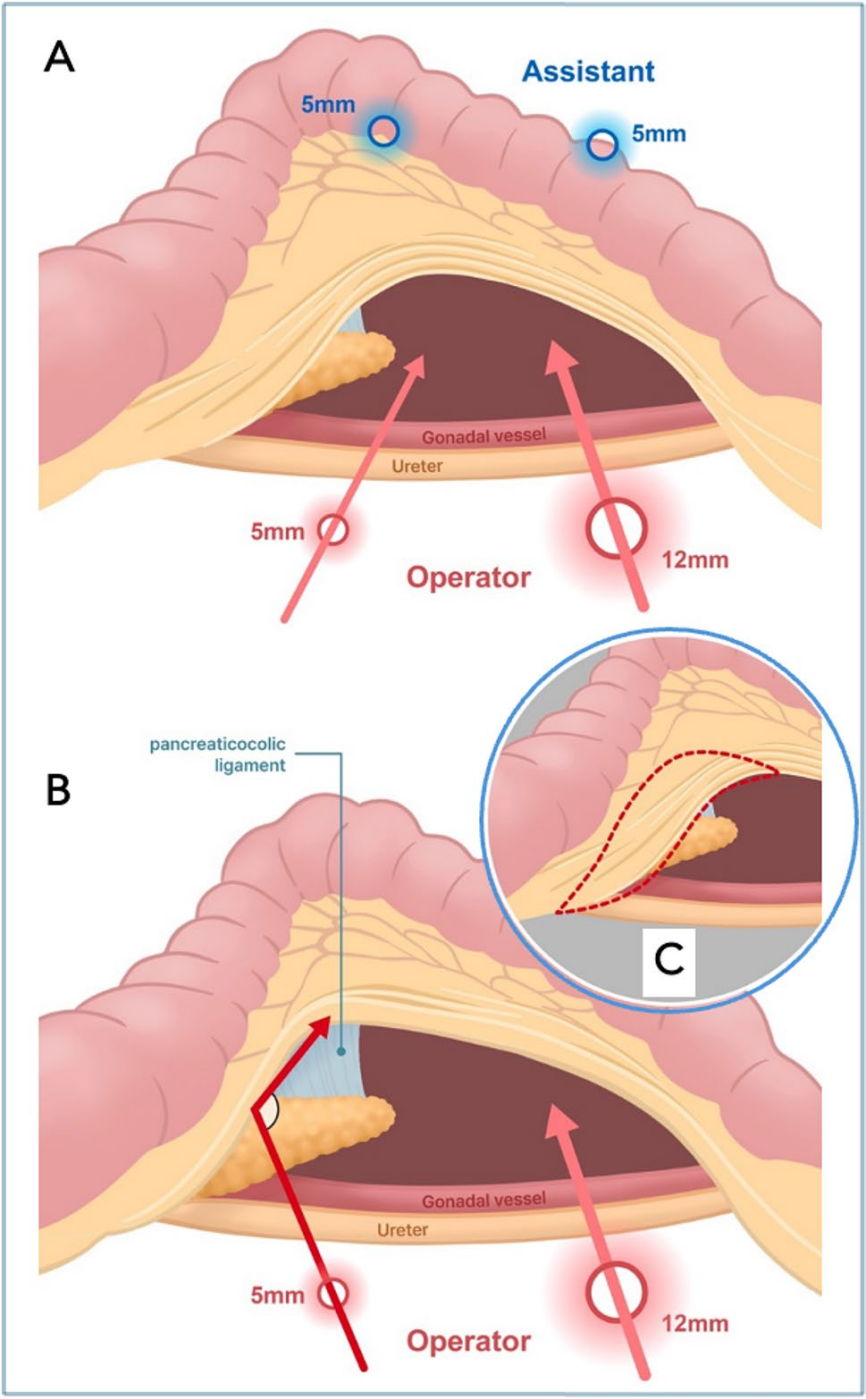
Better exposure of the pancreaticocolic ligament using angulation of the distal joint of the Artisential^®^ grasper. **A** Visualization of the pancreaticocolic ligament utilizing conventional straight laparoscopic instruments. **B** Improved visualization of the pancreaticocolic ligament with articulated instruments. **C** Demarcation of the enhanced view of relevant anatomical structures

In tissue dissection, the dynamic interplay between retraction and tension plays a critical role in facilitating proper visualization of the surgical planes. In this study, we used the exposed area of relevant structures as an indirect marker of this mechanical effectiveness, aiming to objectively represent the technical advantages provided by the ALI. In our experience, the use of Artisential^®^ shifted control of the left colon mesentery from the assistant to the surgeon, allowing for more deliberate and stable retraction. The proximal joint’s angulation enabled efficient lifting of the mesentery, while the distal joint’s flexibility improved visualization of the pancreaticocolic ligament by twisting it into a more favorable plane relative to the laparoscopic view (Fig. 3). This direct surgeon-controlled retraction may have enhanced dissection precision and intraoperative handling, although the study may have been limited in its sample size to detect significant differences in perioperative outcomes.

The average operation time using Artisential^®^ in LARs was comparable to the operations performed without it. Recently, Kang SB et al. [13] reported that at least ten cases were necessary for expert colorectal surgeons to overcome the learning curve in using Artisential^®^ devices for laparoscopic colorectal surgeries. Since the nine LAR cases involving Artisential^®^ in our study occurred within this adjustment period, future LARs performed by the same surgeon utilizing ALIs may achieve even better outcomes as more cases are performed.

Studies indicate that articulated laparoscopic instruments are most beneficial in complex procedures [13–15], as was the case in our practice. Some of these studies also investigated whether such benefits were applicable to surgeons who are novices, and found that they were indeed transferable. In a prospective randomized crossover study on novices using FlexDex, another ALI product, Criss et al. [14] found participants of both groups were able to perform tasks in difficult locations more effectively with an ALI device. The researchers divided the participants into two groups—those with and without training—and found that a key difference between the two groups was not the performance itself, but the higher perceived mental and physical strain reported by those without training. In a related study, Darwich et al. [15] discovered that experts who initially performed peg-transfers significantly faster than novices using an Artisential^®^ grasper, exhibited no significant difference in performance time when a 30-min training session was given for both groups. They also found that all participants, regardless of their levels of expertise, required 8 h of training to surpass their own original performance with a straight grasper. On the basis of these study results, it can be concluded that ALIs improve surgical performance in complex tasks for surgeons of all levels, and that short training sessions can substantially narrow the performance gap between novices and experts. However, achieving proficiency comparable to using conventional devices may require additional hours of training, as previously indicated [13].

We believe our study contributes to the ongoing discussion about ALI in several ways. We introduced the number of harvested lymph nodes as a parameter of specimen integrity to provide a more comprehensive comparison between conventional and articulating instruments. Second, we used low anterior resections performed in two consecutive years by a single surgeon and performed propensity score matching to ensure consistency and minimize selection bias. Third, we aimed not only to describe but also to quantify the added comfort a surgeon experiences when using the Artisential^®^ device—an effort we believe is the first of its kind.

Limitations of this study include its retrospective, nonrandomized design and the limited number of surgeries analyzed. Although propensity score matching was performed to adjust for major clinical variables, body mass index (BMI) and other direct measures of visceral adiposity—which may also affect operative difficulty and outcomes—were not included. A prospective, multicenter study incorporating propensity score matching and adjusting for additional factors such as visceral obesity would further strengthen the evaluation of articulated laparoscopic instrument (ALI) usage.

## Declarations

### Data Availability

No datasets were generated or analyzed during the current study.
